# NFκB in the Development of Endothelial Activation and Damage in Uremia: An In Vitro Approach

**DOI:** 10.1371/journal.pone.0043374

**Published:** 2012-08-22

**Authors:** Carolina Caballo, Marta Palomo, Aleix Cases, Ana M. Galán, Patricia Molina, Manel Vera, Xavier Bosch, Gines Escolar, Maribel Diaz-Ricart

**Affiliations:** 1 Hemotherapy-Hemostasis Department, Centre de Diagnòstic Biomèdic, Institut d'Investigacions Biomèdiques August Pi i Sunyer, Hospital Clinic, Universitat de Barcelona, Barcelona, Spain; 2 Nephrology Department, Institut d'Investigacions Biomèdiques August Pi i Sunyer, Hospital Clinic, Universitat de Barcelona, Barcelona, Spain; 3 Cardiology Department, Institut d'Investigacions Biomèdiques August Pi i Sunyer, Hospital Clinic, Universitat de Barcelona, Barcelona, Spain; Katholieke Universiteit Leuven, Belgium

## Abstract

Impaired hemostasis coexists with accelerated atherosclerosis in patients with chronic kidney disease (CKD). The elevated frequency of atherothrombotic events has been associated with endothelial dysfunction. The relative contribution of the uremic state and the impact of the renal replacement therapies have been often disregarded. Plasma markers of endothelial activation and damage were evaluated in three groups of patients with CKD: under conservative treatment (predialysis), on hemodialysis, and on peritoneal dialysis. Activation of p38 MAPK and the transcription factor NFκB was assessed in endothelial cell (EC) cultures exposed to pooled sera from each group of patients. Most of the markers evaluated (VCAM-1, ICAM-1, VWF, circulating endothelial cells) were significantly higher in CDK patients than in controls, being significantly more increased in the group of peritoneal dialysis patients. These results correlated with the activation of both p38 MAPK and NFκB in EC cells exposed to the same sera samples, and also to the peritoneal dialysis fluids. Hemodialysis did not further contribute to the endothelial damage induced by the uremic state observed in predialysis patients, probably due to the improved biocompatibility of the hemodialysis technique in recent years, resulting in lower cellular activation. However, peritoneal dialysis seemed to exert a significant proinflammatory effect on the endothelium that could be related to the high glucose concentrations and glucose degradation products present in the dialysis fluid. Although peritoneal dialysis has been traditionally considered a more physiological technique, our results raise some doubts with respect to inflammation and EC damage.

## Introduction

Patients with chronic kidney disease (CKD) suffer from complex hemostasis disorders. Both a bleeding tendency and an increased risk of accelerated atherosclerosis, with a high incidence of cardiovascular death, have been described to coexist [1]. Moreover, these patients are known to be exposed to a chronic proinflammatory state and oxidative stress, leading to endothelial cell dysfunction. In hemodialyzed patients, humoral factors such as uremic toxics accumulated in plasma and cytokines released by cellular activation are involved in the development of these pathological processes [2], [3], [4], [5].

The vascular endothelium has been recognized as a complex endocrine organ that regulates many physiological functions such as vascular tone, vascular smooth muscle cell growth and migration, vascular permeability to solutes and blood cells, and regulation of hemostasis, among others [6]. The endothelium is able to adapt to pathophysiological challenges. However, depending on the nature and intensity of the stimuli, the endothelium may become dysfunctional. In this regard, there is clinical [7], [8], [9] and experimental evidence of endothelial activation and damage in uremia [10], [11], [12], [13]. In patients with CKD, the progression of atherothrombosis is accelerated [14], causing early cardiovascular complications [15]. In this regard, mortality from cardiovascular disease is nearly tenfold higher in patients with end-stage renal disease (ESRD) on dialysis than in the general population (US Renal Data System, USRDS 2009 Annual Data Report). This clinical situation cannot be fully explained by an increased prevalence of traditional cardiovascular risk factors such as hypertension, diabetes, hyperlipidemia or smoking, in ESRD [16]. Similarly, an enhanced cardiovascular risk has been reported in patients with CKD not on dialysis [17].

Using endothelial cells in culture, our group has previously characterized the endothelial activation and damage occurring in association with CKD. When exposed to growth media containing sera from patients on hemodialysis, cells showed morphological alterations [13], increased proliferation [13], signs of inflammation with no evidence of apoptosis [12], [13], and an increased thrombogenicity of the generated extracellular matrix [18], [11]. A more recent proteomic approach revealed that there are changes in the expression of some molecules related to inflammation, such as HMGB1 and aldose reductase, and to oxidative stress, such as superoxide dismutase and glutathione peroxidase. These changes were correlated with the activation of the transcription factor NFκB [19].

Most of the studies on the endothelial damage in CKD patients have been conducted in patients undergoing hemodialysis treatment. In the present study, we have investigated the relative contribution of uremia and renal replacement therapies (RRT), hemodialysis and peritoneal dialysis, to the development of endothelial damage in patients with CKD. We applied two different approaches: *ex vivo* analysis of plasma markers of endothelial activation and damage, and *in vitro* evaluation of the signaling mechanisms involved.

## Results

### Main demographic characteristics and biochemical parameters of the patients included in the study

The present studies were carried out in four different groups: i) 15 healthy donors (control group), ii) 11 patients under conservative treatment (PreD group), iii) 15 patients undergoing hemodialysis (HD group), and iv) 9 patients under peritoneal dialysis (PD group). Patients with diabetes and/or dyslipidemia, and smokers were excluded. The main demographic characteristics and biochemical parameters are detailed in Table 1.

**Table 1:** Main demographic characteristics and biochemical parameters.

	Control (n = 15)	PreD (n = 11)	HD (n = 15)	PD (n = 9)
Age, yr, mean±SD	42.3±10.0	62.4±11.2	51.2±14.5	61.4±20.4
Gender male/female	7/8	10/1	8/7	5/4
Mean glomerular filtration rate (ml/min/1.73 m^2^±SD)	–	19.0±7.8	–	–
Residual renal function (ml/min) mean±SD	–	–	0	5.3±4.8
Mean time on dialysis, months±SD	–	–	55.9±70.5	16.1±6.2
Serum Albumin (g/dl) mean±SD	4.2±0.3	4.5±0.2	4.1±0.3	3.7±0.2
Hemoglobin (g/dl) mean±SD	12.2±1.1	12.8±1.8	11.8±1.3	12.1±1.1
Leukocytes (10^9^/l) mean±SD	7.5±1.2	7.6±1.1	6.21±1.96	7.0±1.6
Causes of CKD n (%)				
glomerulonephritis		3 (27.3%)	4 (26.7%)	0
interstitial nephropathy		2 (18.2%)	0	0
renal hypoplasia		1 (0.9%)	0	0
polycystic kidney disease		0	3 (20.0%	2 (22.2%)
systemic lupus erythematosus		0	1 (0.7%)	0
hemolytic uremic syndrome		0	1 (0.7%)	0
bilateral nephrectomy		0	1 (0.7%)	0
obstructive kidney disease		0	1 (0.7%)	0
nephrosclerosis		0	0	2 (22.2%)
unknown		4 (36.4%)	4 (26.7%)	5 (55.6%)
Hypertension n (%)	0	9 (81.8%)	15 (100%)	4 (44.4%)
Use of statins n (%)	0	7 (63.6%)	5 (33.3%)	8 (88.9%)
Use of vitamin D n (%)	0	8 (72.7%)	5 (33.3%)	9 (100%)
Use of erythropoietin n (%)	0	3 (27.3%)	12 (80.0%)	8 (88.9%)
Use of inhibitors of the Renin-Angiotensin System	0	5 (45.5%)	1 (6.7%)	4 (44.4%)

### Levels of endothelial damage markers are increased in dialyzed patients, specially in those under peritoneal dialysis

Plasma sVCAM-1, sICAM-1, and VWF increased significantly in the three groups of patients (p<0.01, p<0.05, and p<0.01 vs. control). Values of sPECAM-1, sE-SELECTIN, and sP-SELECTIN were higher in PreD and PD groups. However, ADAMTS-13 activity was within the normal range (table 2).

**Table 2:** Levels of soluble biomarkers of endothelial activation and damage measured in plasma.

	Control (n = 15)	Predialysis (n = 11)	Hemodialysis (n = 15)	Peritoneal dialysis (n = 9)
sVCAM-1 (ng/ml)	816.9±64.2	1496.0±190.4**	1427.5±191.0**	1781.4±254.1**
sICAM-1 (ng/ml)	130.8±14.0	281.7±15.6*	296.6±20.4*	376.2±35.3*
sPECAM-1(ng/ml)	145.3±21.0	226.7±40.0	155.1±15.3	251.1±40.1*
sE-selectin (ng/ml)	88.8±10.3	109.5±15.8	70.7±7.7	86.1±11.0
sP-selectin (ng/ml)	244.1±22.2	347.6±37.3*	225.3±23.6	331.8±35.4*
VWF (%)	83.6±11.3	153.4±11.4**	210.8±31.9**	173.2±15.7**
CEC/ml	<100	263.3±75.3	155.6±52.2	471.0±119.2*
ADAMTS-13 (%)	103.0±20.0	104.7±6.3	100.1±6.6	93.2±5.1

Data are means±S.E.M.

* p<0.05 and

** p<0.01 vs. the control group.

Circulating EC, identified as CD45−/CD146+/CD31+/CD133+ by flow cytometry, were above the control values (<100 CEC/mL) in all CKD groups, being significantly higher in the PD group with respect to the rest of groups (see table 2).

### Uremic media promotes activation of the p38 MAPK and NFκB p65/ReIA signaling pathways

**Figure 1:**
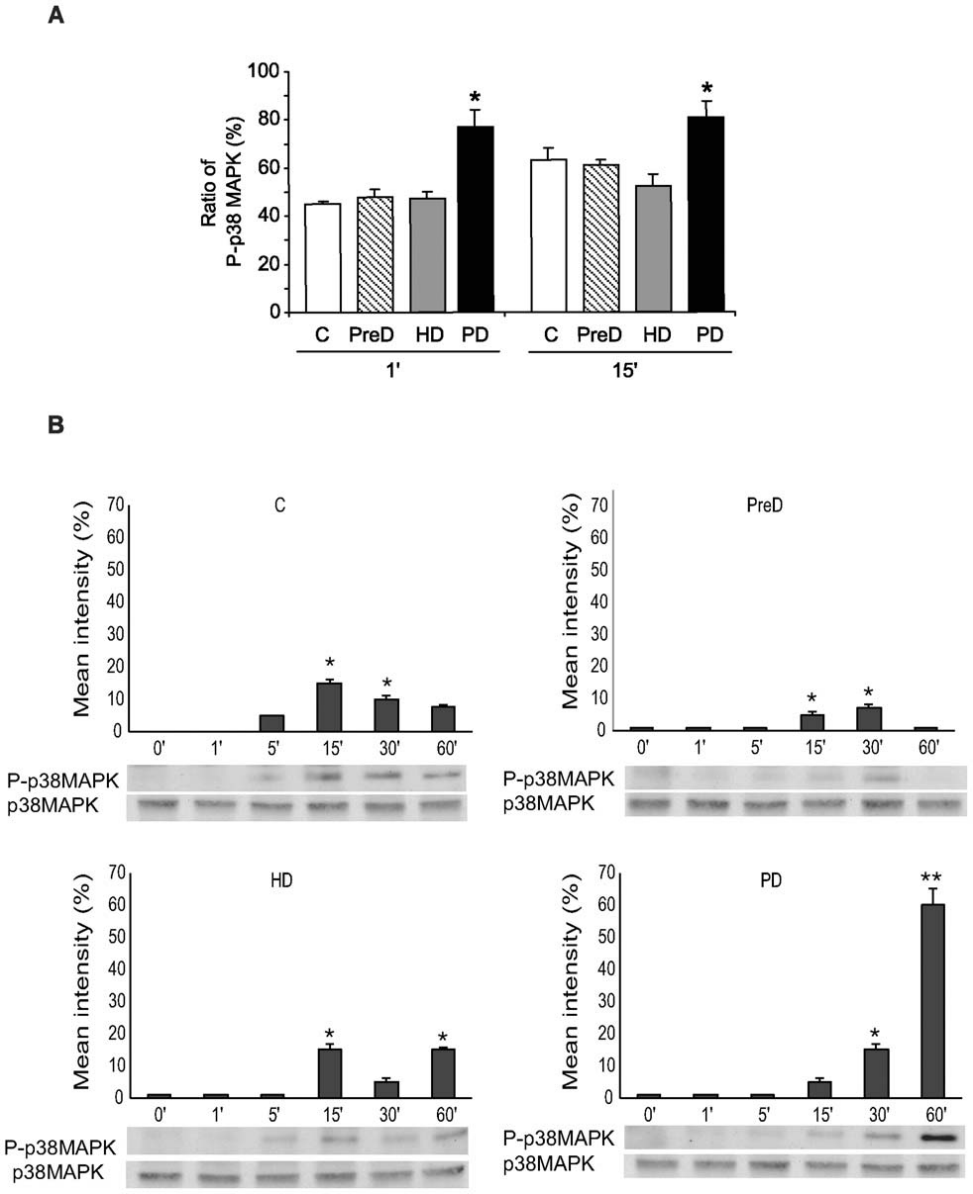
Effect of uremic media on the activation of p38MAPK signaling pathway. **A.** Ratios of phosphorylated p38 MAPK relative to the total protein, obtained in EC after 1 and 15 min of incubation with pooled sera from control donors (C), and predialysis (PreD), hemodialysis (HD), and peritoneal dialysis (PD) groups of patients. Data are corrected for cell number and are represented as mean±SEM (n = 6); *p<0.05 when compared with the other groups. **B.** EC were incubated at different time points with pooled sera from the same groups (C, PreD, HD, and PD, as indicated), and then assayed for phospho-p38 MAPK in cytosolic fractions by Western blot analysis. Images are representative of 6 different experiments and bar diagrams represent the mean intensity of phosphorylation (*p<0.05, **p<0.001).

**Figure 2:**
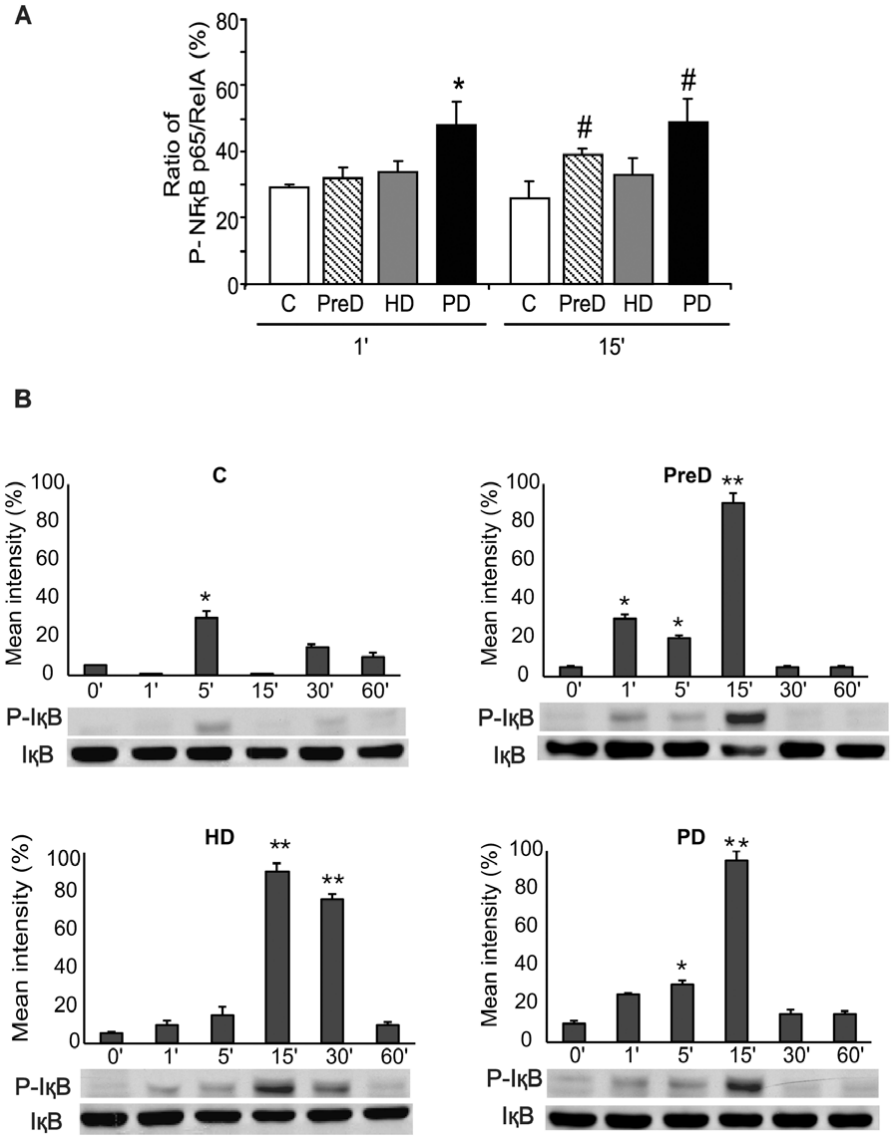
Effect of uremic media on the activation of NFκB p65/ReIA signaling pathway. **A.** Ratios of phosphorylated NFκB p65/ReIA relative to the total protein, obtained in EC after 1 and 15 min of incubation with pooled sera from control donors (C), and predialysis (PreD), hemodialysis (HD), and peritoneal dialysis (PD) groups of patients. Data are corrected for cell number and are represented as mean±SEM (n = 6); *p<0.05 when compared with the other groups; #p<0.05 when compared with the control group. **B.** EC pretreated (for 30 min at 37°C) with N-acetyl-leucyl-leucyl-norleucinal (ALLN, 50 µg/ml) were incubated at different time points with pooled sera from the same groups (C, PreD, HD, and PD, as indicated), and then assayed for phospho-IκBα in cytosolic fractions by Western blot analysis. Images are representative of 6 different experiments and bar diagrams represent the mean intensity of phosphorylation (*p<0.05, **p<0.001).

Changes in the activation state of both p38 MAPK and NFκB, expressed as the relative extent of the phosphorylated target protein, are shown in figure 1 and 2, respectively. Two different techniques were applied: a phosphospecific antibody cell-based ELISA, which is faster but probably less sensitive, and immunoblotting as a reference method.

In relation to p38 MAPK, a statistically significant increase in the levels of the phosphorylated protein was observed in cells exposed to sera from the PD group, after 1 and 15 min (increases of 71.4±2.5% and 28.4±1.9% vs. control, respectively, both p<0.05, n = 6). Levels of p38 MAPK activation in the PreD and HD groups were similar to those observed in the control group. Increases of 7.4±0.3% and 4.2±0.01% in response to PreD and HD sera, respectively, were detected after 1 min of activation, and no significant variations were detected after 15 min. Activation of p38 MAPK in cells exposed to the conditions under study was confirmed by immunoblotting (see figure 1). Although activation of p38 MAPK was observed in the C, PreD and HD groups, specially at 15 min, it was mild. Phosphorylation of p38 MAPK increased progressively when cells were exposed to sera from the PD group from 15 to 60 min, being really significant at 60 min (increase of the mean intensity from 0 to 60%, p<0.001).

Additionally, NFκB p65/ReIA showed a higher ratio of phosphorylation when EC were exposed to the sera of the three groups of uremic patients when compared to the control group. The most notable changes were observed when EC were incubated with the sera from PD patients at 1 and 15 min of exposure (increases of NFκB activation of 65±2.9% and 85±2.6% vs. control, respectively, both p<0.05, n = 6). Although not statistically significant, there was also an activating effect of the HD sera (increases of 19±4.5% and 27±3.8% at 1 and 15 min of exposure, n = 6). Sera from PreD patients also caused a significant activation of NFκB at 15 min (46±2.1%, p<0.05 vs. control, n = 6).

Western-blot analysis of phospho-IκB showed that this transcription factor was equally activated in cells exposed to the sera from the three groups of patients and it was not detected in cells exposed to control sera (see figure 2). Mean intensity of phosphorylation in cells exposed to sera from the three different groups of uremic patients was maximal at 15 min of exposure (p<0.001) and was sustained till 30 min in the HD group (p<0.001).

### Effect of the glucose and its degradation products on endothelial damage and inflammation

The results obtained throughout the present study led us to explore the potential causes of endothelial activation in the group of peritoneal dialysis patients. Glucose degradation products (GDP) present in heat-sterilized dialysis solutions are thought to contribute to cellular dysfunction and membrane damage during peritoneal dialysis. Therefore, we examined the impact of conventional and low GDP peritoneal dialysis solutions with different concentrations of glucose (1.5%, 2.3%, and 4.25%), and Icodextrin, on the activating state of the signaling molecules explored.

**Figure 3:**
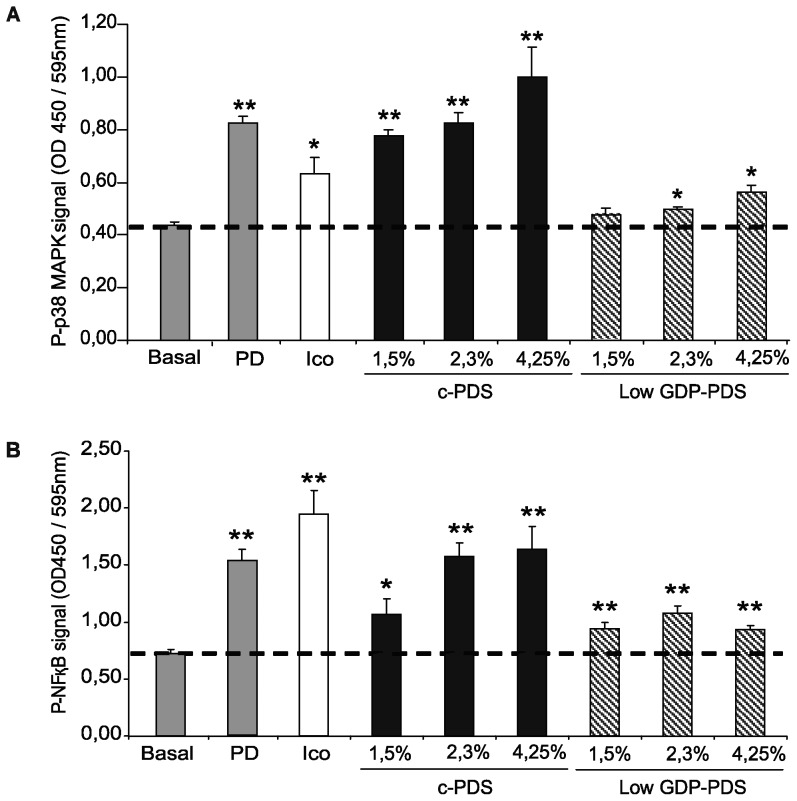
Effect of different peritoneal dialysis fluids on the activation of p38MAPK and NFκB p65/ReIA signaling pathways. Levels of phosphorylated p38 MAPK (A) and NFκB p65/ReIA (B), represented as phospho-p38 MAPK and phospho-NFκB signal at 450/595 nm, were evaluated in EC after 15 min of incubation with the pooled sera from the PD group and with the different peritoneal dialysis solutions: icodextrin (Ico) (7.5%), conventional (c-PDS) and low GDP (Low GDP-PDS) peritoneal dialysis solutions (at the concentrations of glucose of 1.5%, 2.3%, and 4.25%). Data were plotted after correction for cell number and are represented as mean±SEM (n = 6); *p<0.05 and **p<0.01 when compared with basal levels of phosphorylation.

Phosphorylation of both p38 MAPK and NFκB p65/ReIA, expressed as the phosphorylation signal measured at 450 nm and corrected by the relative number of cells measured at 595 nm, was really significant in cells exposed to all the solutions tested for 15 min (see figure 3).

When compared to the basal condition, phosphorylation levels of p38 MAPK increased 85±2.5% (p<0.01) in response to the PD sera, and 44±2.8% (p<0.05) in response to icodextrin. When cells were exposed to the 1.5%, 2.3% and 4.25% c-PDS, phosphorylation of p38 MAPK was more notable and occurred in a concentration-dependent manner, increasing in 76±2.3%, 87±3.4% and 127±5.6% (all p<0.01). The activating effect of the low GDP-PDS, although significant vs. the basal state and in a concentration dependent manner, was always below that induced by the PD pool of sera (increases of phosphorylation of 9±1.9%, 13±0.9% and 28±2.5%, p<0.05, in response to the 1.5%, 2.3% and 4.25% GDP-PDS vs. the basal state).

In relation to the the NFκB, results were similar to those observed for p38 MAPK. Activation vs. the basal state increased to 114±3.0% with PD sera, 53±3.2%, 125±3.1% and 134±4.0% in response to the 1.5%, 2.3% and 4.25% c-PDS, and 35±1.8%, 54±1.7% and 34±0.9% in response to the 1.5%, 2.3% and 4.25% GDP-PDS. The effect of Icodextrin was much more intense on the phosphorylation of the transcription factor NFκB, even above all the conditions studied (increase of 178±4.9% vs. the basal state).

## Discussion

The present study was focused to discern the contribution of uremia and the RRT to the development of endothelial activation and damage. The *ex vivo* and *in vitro* approaches applied revealed that uremia *per se* causes a proinflammatory state on the endothelium, as derived from results in pre-dialysis patients. The hemodialysis technique with the current advances (generalized use of biocompatible membranes and ultrapure water, among others) did not exhibit a damaging effect on the endothelium additionally to that observed in patients with advanced CKD managed conservatively. Interestingly, peritoneal dialysis was the most proinflammatory condition, as demonstrated by a higher presence of soluble markers of endothelial activation and damage in plasma, and a more intense activation of both p38 MAPK and NFκB signaling pathways in cultured endothelial cells. These effects could be attributed, at least in part, to the glucose and its degradation products present in the PD dialysis fluids.

Endothelial activation and damage could be the earliest indicator of subclinical cardiovascular disease. In the laboratory, measurement of plasma levels of different molecules and/or other elements, either discharged or up-regulated in an activated endothelium, could be useful to assess endothelial damage and activation [20], [21]. Considering that to date there is no universal marker of endothelial damage, we have evaluated different indicators. Soluble adhesion receptors were evaluated by a multiplex system and showed significant activation of the endothelium in all uremic patients, being more notable in the PreD and PD groups. In relation to VWF plasma levels, our present data is consistent with previous observations in hemodialyzed patients [7] being significantly higher in all the uremic patients than in controls. However, the short range in which values are included might explain the lack of differences found between the studied groups. Levels of circulating endothelial cells (CEC) have been described to correlate well with VWF plasma levels [22]. There is previous evidence generated in HD patients of increased CEC and their association with future cardiovascular events [10], [23]. In our present study, blood CEC counts were higher in all the uremic patients when compared to the control group, especially in the group of peritoneal dialysis.

The analysis of plasma markers of endothelial damage indicates that the toxic environment of uremia causes inflammation on the endothelium, as observed in the non dialyzed CKD patients. This inflammatory effect is also seen in patients under both types of RRT. From the results obtained, it can be predicted that PD exhibits the most deleterious effect on the endothelium and, interestingly, current HD techniques have diminished the harmful effect previously ascribed to this treatment.

Previous studies by our group and others were mostly performed in hemodialyzed patients. Results have demonstrated the existence of endothelial activation and damage in CKD both *in vivo* and *in vitro*. Plasma levels of endothelium-derived proteins [7], some of them vasoactive factors, are increased in these patients [24]. Endothelium-dependent vasodilation, the gold-standard method to assess endothelial function *in vivo*, is also decreased in these patients [25]. Exposure of cultured EC to sera from hemodialyzed patients accelerates cell cycle and proliferation, with a more prothrombotic extracellular matrix and a proinflammatory phenotype with a higher expression of cell adhesion molecules [13], [12], which represents one of the earliest pathological changes in immune and inflammatory diseases such as atherosclerosis [26]. Moreover, a proteomic characterization of these changes demonstrated a differential expression of proteins associated with inflammation and oxidative stress in cells exposed to the uremic condition, related to the NFκB signaling pathway [19]. In fact, exposure of endothelial cells in culture to sera from hemodialyzed patients induces activation of p38 MAPK [12] and of NFκB [19]. In hemodialyzed patients there is presence of the uremic toxics but also of those components released by blood cells that become activated by the procedure itself. However, results from the present study indicate that there is not an additional deleterious effect of hemodialysis over that observed in the predialysis condition. Probably, the generalized use of more biocompatible dialysis membranes and ultrapure water, among other advances, has reduced blood cell stress with less contribution of proinflammatory cytokines. Interestingly, PreD patients showed a more significant inflammatory state than HD patients. These results are not fully in agreement with those by Merino et al. [27]. In their study, PD seems to exhibit a lower damaging condition on the endothelium than HD and predialysis. Differences between both studies may be due to the different experimental approaches applied but especially to the fact that in our study patients with CKD did not have evidence of previous cardiovascular disease and other known cardiovascular risk factors.

The present study provides the first evidence that sera from PD patients have a greater activating effect on p38 MAPK and NFκB, two intracellular key markers of inflammation and cell damage. The p38 MAPK protein kinases affect a variety of intracellular responses, with well-recognized roles in inflammation, cell-cycle regulation, cell death, development, differentiation, senescence and tumorigenesis [28]. NFκB seems to act by regulating the expression of several genes involved in tumorigenesis, including anti-apoptotic proteins, cyclooxygenase-2, matrix metalloproteinase-9, genes encoding adhesion molecules, chemokines, and inflammatory cytokines; and cell cycle regulatory genes [29]. Therefore, both p38 MAPK and NFκB participate in the proinflammatory responses and exhibit a clear role in the development of inflammatory and immunological diseases.

According to the USRDS 2011 Annual Data Report, mortality rates of dialyzed patients have declined in the last years probably reflecting changes in catheter utilization, improved cardiovascular disease care, and changes in infectious complications. However, cardiovascular disease is still the major cause of the limited life expectancy in patients on substitutive therapies [30], [31].

Patients under peritoneal dialysis are often hypertensive and sometimes volume overloaded [32], which is related to endothelial damage in PD patients [33]. Glucose is absorbed to a large extent from the dialysate, and conventional PD results in an almost unique metabolic situation involving continuous 24-hour absorption of glucose. One common and important side effect of this treatment is weight gain and accumulation of body fat stores [34], [35]. These patients develop hyperlipidemia, with high levels of low-density lipoprotein and triglycerides. Advanced glycation end-products (AGEs), which are believed to promote atherosclerosis through interaction with endothelial receptors [36], are commonly accumulated in CKD patients due to decreased renal clearance, could be overproduced in PD patients due to the high exposure to glucose and glucose degradation products (GDP). Bioincompatibility of conventional glucose-based peritoneal dialysis fluids (PDF) has been partially attributed to the presence of GDP generated during heat sterilization of PDF [37], [38], which are thought to contribute to cellular dysfunction and membrane damage during peritoneal dialysis [39], [35], [40]. In accordance with these previous results, our present study shows that the glucose load and the presence of GDP could play a role in the development of endothelial damage among these patients. Icodextrin is a colloid osmotic agent, derived from maltodextrin, used as aqueous solution for peritoneal dialysis. The osmotic activity of icodextrin keeps the solution inside the peritoneum for 10 to 16 hours without being significantly metabolized. Due to its chemical characteristics, Icodextrin reduces the burden of glucose overexposure. From this perspective, the activating effect of Icodextrin on the transcription factor (and to a lesser extent on the inflammation-dependent protein p38 MAPK) is difficult to explain. It can however not be excluded that the pH of the Icodextrin solution could exert a damaging effect on the endothelium in our in vitro studies.

From our present results it can be concluded that there is endothelial activation and damage associated with CKD, as demonstrated by the increased presence of plasma markers and by the *in vitro* studies in cultured endothelial cells. The uremic state seems to be a major cause of endothelial damage, probably through the activation of transcription factors, such as NFκB, which are related to inflammation. However, while improved hemodialysis procedures do not seem to have an additional deleterious effect, our different experimental approaches applied indicate that peritoneal dialysis seems to exert a more intense proinflamatory action on the endothelium that could be due, at least in part, to the increased glucose load. Studies to elucidate the potential molecular mechanisms involved should be the aim of future investigation.

## Materials and Methods

### Ethics statement

Written informed consent was obtained for blood utilization from every healthy donor and patient included in the study. The study was approved by the ethical committee of the Hospital Clinic (2011/6238) and was carried out according to the principles of the Declaration of Helsinki.

### Experimental Design

We have applied both an *ex vivo* approach to analyze the presence of soluble plasma biomarkers of endothelial activation and damage and an *in vitro* approach to explore the activation of key signaling pathways related to inflammation in cultured endothelial cells exposed to sera from uremic patients.

In order to investigate the relative contribution of uremia and substitutive therapies, studies were carried out in four different groups: i) 15 healthy donors (control group), ii) 11 patients under conservative treatment (PreD group), iii) 15 patients undergoing hemodialysis (HD group), and iv) 9 patients under peritoneal dialysis (PD group). Patients with diabetes and/or dyslipidemia, and smokers were excluded.

In subsequent experiments, endothelial cells were exposed to conventional and low GDP peritoneal dialysis solutions with different concentrations of glucose (1.5%, 2.3%, and 4.25%), and to Icodextrin (7.5%), to explore their effect on the signaling pathways under study.

The present study has some limitations, mainly due to its nature: this is a cross-sectional observational study in which the sample population is small. However, the ex vivo measurements were performed in duplicate and confirmed by different techniques. The in vitro studies were performed in triplicate, using pooled sera from each group of patients to minimize variations, and also confirmed by two different experimental approaches.

### Patients and sample collection

PreD samples were obtained from 11 (7 men and 4 women) non dialyzed patients with CKD (stage IV and V), age 62.4±11.2 (mean±SD), glomerular filtration rate 19.0±7.8 ml/min/1.73 m^2^, with glomerulonephritis (3), interstitial nephropathy (3), renal hypoplasia (1), and unknown (4). HD group consisted of 8 men and 7 women, age 55.2±14.5, residual renal function 0 ml/min, and time on dialysis 55.9±70.5 months. Causes of renal failure in these patients were glomerulonephritis (4), polycystic kidney disease (3), systemic lupus erythematosus (1), hemolytic uremic syndrome (1), bilateral nephrectomy (1), obstructive kidney disease (1), and unknown (4). All HD patients were dialyzed with biocompatible membranes, ultrapure water, for ≥4 hours, with a Kt/V≥1.3. Samples were always obtained before the HD session. In the PD group, 5 men and 4 women with polycystic kidney disease (2), nephrosclerosis (2), and unknown (5) were included, age 61.4±20.4, residual renal function 5.3±4.8 ml/min, and time on dialysis 16.1±6.2 months. PD patients only used biocompatible solutions with low levels of GDP: 5 Bicavera™ (Fresenius, Spain), 2 Physioneal™ (Baxter, Spain), 2 Gambrosol Trio™ (Gambro, Spain) and 3 patients used Extraneal™ (Baxter, Spain). The glucose concentration used or the indication for icodextrin (Extraneal™) was based on the patient's hydration status and peritoneal transport characteristics. None of the PD patients had had a peritonitis episode in the previous 2 months. See table 1 for patients data.

Diabetic patients or patients with dyslipidemia and/or previous cardiovascular disease and smokers were excluded. Most of the patients had no vascular disease as a cause of CKD. Samples from healthy donors were obtained as controls (n = 15, 7 male and 8 women, mean age 61.4±20.4).

Plasma and sera samples were obtained from each patient by centrifugation of citrated blood (800×g, 15 min) and non anticoagulated blood (3000×g, 15 min), respectively, and stored at −20°C until use.

### Analysis of soluble markers of endothelial activation and damage

Plasma levels of VCAM-1, ICAM-1, PECAM-1, E-SELECTIN and P-SELECTIN were quantified by flow cytometry (Flowcytomix, Bender MedSystems; LabClinics S.A, Barcelona, Spain). This system employs capture antibodies coupled to fluorescent polystyrol beads of different sizes. A dual-laser flow cytometer (FACScan, Becton-Dickinson, Mountain View, Ca, USA) identifies the beads based upon their size and fluorescence intensity, and then quantifies the amount of antigen by measuring fluorescence emitted from Streptavidin-Phycoerythrin (PE) associated with a biotinylated detector antibody.

Circulating EC were measured by the flow cytometry method, in which whole blood was labelled with monoclonal antibodies tagged with fluorochromes (anti-CD45-PerCP, anti-CD146-FITC, anti-CD31-PE, anti-CD133-APC) (Chemicon Int, BD Pharmingen, Miltenyi Biotech, Becton & Dickinson).

VWF was evaluated by ELISA (American Diagnostica, Grifols SA, Barcelona, Spain). ADAMTS-13 activity was analyzed in citrated plasma by a fluorescence resonance energy transfer assay using a truncated synthetic 73-amino-acid VWF peptide as a substrate (FRETS-VWF73 assay) [41].

### Culture of human umbilical vein endothelial cells

Primary cultures of EC were isolated from human umbilical cords veins, according to a previously described method [42], by collagenase treatment (0.2% in phosphate buffered saline, PBS, 15 min, 37°C) (Boehringer Mannheim, Mannheim, Germany). Cells were grown with culture medium (Mem 199; Gibco BRL, Life Technologies, Scotland, UK) supplemented with 100 U/ml penicillin, 50 mg/ml streptomycin and 20% pooled human serum. EC were grown at 37°C in a 5% CO_2_ humidified incubator. The culture medium was changed every 48 h. After the second passage, EC were subcultured for 24 h on 0.1% gelatin-coated 96-well plates or on 0.1% gelatin-coated 6-well plates. Cells were used before reaching confluency.

### Activation of the p38 MAPK and NFκB signaling pathways in endothelial cells

The activation of the p38 MAPK and NFκB p65/ReIA signaling pathways was assessed in cultured EC by two methods: a phosphospecific antibody cell-based ELISA, and immunoblotting.

To assess activation of p38 MAPK and NFκB by the phosphospecific antibody cell-based ELISA, endothelial cells were seeded in 96-well plates. After 24 h, confluent cells were serum starved during 4 h before experiments were performed by replacing their growth media with media containing 2% pooled human serum. This experimental procedure ensures basal levels of protein activity. Cells were then incubated for 1, 5, and 15 min with the 4 different pooled sera. The activation (phosphorylation) was evaluated by the cellular activation of signaling ELISA (CASE) kit superArray (SABiosciences, Tebu-bio Lab, Spain). The kit includes a complete antibody-based detection system for colorimetric quantification of the relative amount of phosphorylated protein and total target protein [43]. Detection of total and phosphorylated protein expression was determined according to the manufacturer's protocol and the levels obtained were corrected for cell number.

The activation of each of these signaling pathways was confirmed by immunoblotting as a reference method. EC were lysed with Laemmli buffer (125 mmol/l of Tris-HCl, 2% SDS, 5% glycerol, and 0.003% bromophenol blue), sonicated for 15 seconds to shear DNA and reduce viscosity, and heated to 90°C for 5 min. Protein concentrations of the supernatants were determined using Coomassie Plus (Pierce) as recommended by the manufacturer [44]. Samples were resolved by using 8% SDS-PAGE and proteins were transferred to nitrocellulose membranes, which were probed with specific antibodies to p38 MAPK phosphorylated at Thr180 and Tyr182, and to phosphorylated IκB (Cell Signaling Technology Inc, Frankfurt, Germany). Considering that translocation of NFκB to the nucleus is preceded by the phosphorylation and degradation of IκB, we analyzed the phosphorylation of the IκB protein in the presence of N-acetyl-leucyl-leucyl-norleucinal (ALLN) which prevents its degradation by the 26S proteasome [45]. Then, membranes were incubated with a peroxidase-conjugated anti-rabbit immunoglobulin G and developed using the chemiluminiscence technique. The presence of proteins was confirmed using specific antibodies. Densitometric analysis was performed to quantify the intensity of phosphorylation (ImageJ, National Institutes of Health, Bethesda, Maryland, USA).

### Effect of the peritoneal dialysis fluids on p38 MAPK and NFκB signaling pathways

The effect of Icodextrin (Extraneal, Baxter, Spain), conventional peritoneal dialysis solutions (Stay-Safe 1.5%, Stay-Safe 2.3%, Stay-Safe 4.25% from Fresenius Medical Care, Spain), and low GDP peritoneal dialysis solutions (BicaVera 1.5%, BicaVera 2.3%, BicaVera 4.25% from Fresenius Medical Care, Spain) on the activation of p38 MAPK and NFκB in cultured endothelial cells was also assessed.

The amount of activated (phosphorylated) p38 MAPK and NFκB p65/ReIA was quantified in cultured endothelial cells after 15 min of incubation by using the Cellular Activation of Signaling ELISA (CASE™) kit superarray (SABiosciences, Tebu-bio Lab, Spain).

Cell viability was assessed in cultures exposed to the solution under study by the uptake of the vital dye Trypan blue (0.4% in PBS), according to standardized protocols for cell culturing and maintenance, and also by the MTT method. Cell viability was never lower than 90%.

### Statistics

Results are expressed as mean ± standard error of the mean (SEM) and as ratios of phosphorylated protein with respect to the total protein values. Statistical analysis was performed with raw data using the Student's t-test for unpaired samples and ANOVA. Results were considered statistically significant when p<0.05. The SPSS statistical package 17.0.0 (SPSS Inc, Chicago, IL) was used for all analyses.
